# Comparative Analysis of Volatile Compounds, Amino Acids, Fatty Acids, and Lipidomic Profiles in Thigh Muscles of Commercial Arbor Acres (AA) Broilers and Indigenous Chengkou and Langshan Chickens

**DOI:** 10.3390/foods15173098

**Published:** 2026-08-31

**Authors:** Xuan Hu, Yanfeng Fan, Qian Zhou, Xiaoxu Jia, Jing Zhang, Xiaohui Feng, Lina Ma, Yinyin Liu, Yushi Gao, Xiujun Tang

**Affiliations:** 1 Jiangsu Institute of Poultry Science, Yangzhou 225009, China2023405004@stu.njau.edu.cn (X.J.); marina1986tiger@163.com (L.M.); 18901456756@163.com (Y.L.); 2 College of Veterinary Medicine, Yangzhou University, Yangzhou 225009, China; 3 Institute of Animal Sciences, Chinese Academy of Agricultural Sciences, Beijing 100193, China

**Keywords:** chicken breed, lipidomics, volatile compounds, amino acids, fatty acids

## Abstract

Flavor-related compounds and nutritional components of chicken meat vary among different breeds, but comprehensive comparisons of these characteristics between commercial and indigenous chickens remain insufficiently characterized. In this study, three chicken breeds (Arbor Acres, Chengkou, and Langshan) were slaughtered at their respective market ages, and the volatile flavor compounds, amino acids, fatty acids, and lipidomic profiles of thigh muscle were analyzed to investigate breed-associated differences in flavor-related and nutritional characteristics. Langshan chickens exhibited the highest total volatile compound content and also had the highest total amino acid levels, with significantly higher contents of umami and sweet amino acids. In addition, both indigenous breeds showed higher levels of arachidonic acid (C20:4n6) than Arbor Acres broilers, while Chengkou chickens had the highest content of docosahexaenoic acid (DHA, C22:6n3). Lipidomic analysis identified 787 lipids, with glycerophospholipids and sphingolipids as the predominant classes. Differential lipid analysis revealed that Langshan chickens had 38 upregulated lipids compared with Arbor Acres chickens, while Chengkou chickens exhibited 258 differential lipids relative to Arbor Acres chickens. Kyoto Encyclopedia of Genes and Genomes (KEGG) pathway enrichment analysis indicated that these differential lipids were mainly associated with glycerolipid, sphingolipid, and glycerophospholipid metabolism. Correlation analysis further revealed significant associations between specific lipids and flavor-related compounds, amino acids, and fatty acids, suggesting their potential roles in breed-associated differences. Overall, this study demonstrates that indigenous chicken breeds possess distinct flavor-related and nutritional profiles compared with commercial Arbor Acres broilers and provides valuable insights into breed-associated differences in chicken meat characteristics.

## 1. Introduction

Chicken meat is widely consumed worldwide and is recognized as an important source of high-quality protein in human diets. Compared with red meat, chicken generally contains lower levels of fat, saturated fatty acids, and cholesterol, making it a nutritious animal-derived food [1,2]. With improving living standards, consumer demand for high-quality poultry products has grown significantly. In China, one of the world’s largest producers and consumers of chicken meat, dietary preferences have gradually shifted from quantity-oriented consumption to quality-oriented consumption.

Consequently, premium local chicken breeds such as Langshan and Chengkou Mountain chickens have attracted attention for their distinctive flavor and desirable muscle texture, indicating considerable market potential. Chengkou Mountain chicken, an excellent indigenous breed, is known for its strong adaptability to extensive feeding, desirable meat quality, disease resistance, and high nutritional value [3,4]. Langshan chickens are mainly raised in Rudong County, Nantong City, Jiangsu Province, whereas Chengkou Mountain chickens are primarily produced in Chengkou County in the Daba Mountain region of Chongqing. In contrast, Arbor Acres (AA) broiler is a widely used commercial white-feathered breed selected for fast growth and high feed efficiency. While previous studies have reported differences in meat quality characteristics between indigenous chickens and commercial broilers [5,6], the molecular mechanisms underlying the unique flavor profiles of Langshan and Chengkou Mountain chickens remain understudied.

A more comprehensive understanding of meat quality requires moving beyond traditional sensory evaluation toward molecular-level characterization of key meat constituents. Flavor is a critical determinant of food quality and plays a decisive role in consumer acceptance [7]. Flavoromics, a non-targeted branch of metabolomics, facilitates the identification of key aroma compounds and helps link food composition with sensory properties [8,9]. Amino acids not only contribute to nutritional value but also serve as essential precursors of volatile flavor compounds via the Maillard reaction and Strecker degradation [10]. Lipids are likewise crucial, as they influence both sensory and nutritional characteristics, including unsaturated fatty acid composition and the levels of fat and cholesterol [11,12]. In addition, lipids are involved in fundamental biological processes such as metabolism, cell signaling, and membrane integrity [13]. Recent studies have suggested that specific lipid species, including fatty acids and triacylglycerols, may serve as discriminative biomarkers for chicken breeds or tissue types [12]. Meanwhile, advances in gas chromatography-mass spectrometry (GC-MS) and liquid chromatography high-resolution mass spectrometry (LC-HRMS) have greatly facilitated the comprehensive profiling of flavor compounds, amino acids, and lipids. Integrated approaches combining flavoromics, amino acid profiling, and lipidomics have been increasingly applied in meat science to elucidate the molecular basis of meat quality [14]. Nevertheless, systematic multi-omics studies characterizing the distinctive quality attributes of indigenous chicken breeds remain limited.

To address this gap, this study focused on two representative Chinese indigenous black-feathered breeds, Chengkou Mountain and Langshan chickens, as well as commercial white-feathered AA broilers. We systematically evaluated their amino acid profiles, intramuscular fat content, fatty acid composition, volatile flavor compounds, and lipidomic signatures. Integrating these analyses, we aimed to identify breed-specific nutritional and flavor-related molecular biomarkers, laying a molecular basis for the high-value utilization of indigenous chicken germplasm and the development of refined meat quality evaluation standards.

## 2. Materials and Methods

### 2.1. Animals

All animal procedures were approved by the Animal Ethics Committee of the Jiangsu Institute of Poultry Science (Permit No.: JIPS202404019, Jiangsu, China). The experimental design strictly conformed to the 3R principles (Replacement, Reduction, Refinement) of animal welfare ethics, and all procedures adhered to the NIH Guide for the Care and Use of Laboratory Animals (8th edition) and the Chinese national standard GB/T 35892–2018. A total of 360 healthy female chickens (120 birds per breed) were reared according to the conventional production practices of each breed and harvested at their respective commercial market ages: 17 weeks for Langshan and Chengkou Mountain chickens and 6 weeks for Arbor Acres (AA) broilers. These slaughter ages corresponded to the routine production cycles and commercial market ages of the respective breeds. From this larger cohort, 30 birds with comparable body weights were randomly selected from each breed (90 birds in total) for the present study. The number of biological replicates varied depending on the analytical method. Six biological replicates per breed were used for amino acid, fatty acid, and lipidomic analyses, whereas eight biological replicates per breed were used for volatile compound analysis. All birds were housed in an environmentally controlled poultry house with consistent lighting, ventilation, and hygiene conditions throughout the rearing period. The three breeds were reared simultaneously under the same environmental conditions, with separate pens assigned to each breed to avoid mixing. They had ad libitum access to fresh drinking water. The dietary composition and nutritional profiles of the three chicken breeds are detailed in Supplementary Table S1. The experimental chickens were sourced from specialized genetic resource institutions as follows: Langshan chickens from the Rudong Langshan Chicken Conservation Farm; AA broilers from the National Poultry Performance Testing Station (Yangzhou, China); and Chengkou Mountain chickens from the Chengkou Mountain Chicken Genetic Resources Institute.

### 2.2. Sample Collection

All chickens were electrically stunned at an oscillating frequency of 900 Hz for 5 s (40 V) using a poultry stunning device (SQ05A, Aneng, Wujiang Aneng Electronic Technology Co., Ltd., Suzhou, China), followed by exsanguination. Chickens were fasted for 12 h before slaughter with free access to water. After slaughter, thigh muscle samples were immediately collected from each chicken, snap-frozen in liquid nitrogen, and stored at −80 °C. These stored samples were used to analyze volatile flavor compounds, amino acid composition, fat content, fatty acid composition, and lipidomic profiles.

### 2.3. Volatile Flavor Compound Analysis

Volatile flavor compounds were extracted and analyzed using headspace solid-phase microextraction coupled with gas chromatography-high-resolution mass spectrometry (HS-SPME-GC-HRMS) according to the method described by Yang et al. [15], with the following parameters. Briefly, 2.5 g of homogenized thigh muscle was placed in a headspace vial, and 10 μL of 2-methyl-3-heptanone (10 μg/mL) was added as an internal standard. The vial was sealed and heated at 85 °C for 30 min. A 50/30 μm DVB/CAR/PDMS fiber was conditioned at 270 °C for 15 min, then exposed to the sample headspace at 55 °C for 40 min after 20 min of equilibration. Desorption was performed at 250 °C for 3 min. Chromatographic separation was carried out on a VF-WAX MS capillary column (60 m × 0.25 mm i.d. × 0.25 μm, Agilent Technologies, Santa Clara, CA, USA) using a Trace 1310 GC system (Thermo Fisher Scientific, Rodano, Milan, Italy). Helium (99.99%) was used as the carrier gas at a constant flow rate of 1.0 mL/min in splitless mode. The oven temperature was programmed as follows: initially 40 °C, held for 2 min, then increased at 4 °C/min to 230 °C, and held for 5 min. The injector temperature was set at 250 °C. Mass spectrometry was performed with a Q-Exactive Orbitrap MS (Thermo Fisher Scientific) operating in full-scan mode (*m*/*z* 30–400) at a resolution of 60,000 under electron impact ionization (70 eV). The ion source and transfer line temperatures were set at 280 °C and 250 °C, respectively. Data acquisition and processing were performed using Xcalibur 4.1 and TraceFinder 4.0 software. Volatile compounds were identified by comparing mass spectra and linear retention indices with the NIST17 database and an in-house library. Linear retention indices were calculated using a series of n-alkanes (C7–C40). Quantification was performed using the internal standard method based on peak area ratios.

### 2.4. Amino Acid Composition Analysis

Amino acid contents were determined using an amino acid analyzer (LA 8080, Hitachi, Tokyo, Japan) according to the national standard GB 5009.124-2016. Briefly, samples were hydrolyzed with 6 mol/L HCl at 110 °C for 22 h under a nitrogen atmosphere. After hydrolysis, the solution was evaporated to dryness, reconstituted in sodium citrate buffer (pH 2.2), and filtered through a 0.22 μm membrane. Separation was performed on a sulfonic acid cation-exchange resin column, and detection was carried out at 570 nm (440 nm for proline) after post-column ninhydrin derivatization. Amino acids were quantified by the external standard method.

### 2.5. Fat Content Analysis

Fat content was determined using a Soxtec™ 8000 automatic fat extraction system (FOSS, Hillerød, Denmark) according to GB 5009.6-2016. Briefly, 2 g of homogenized thigh muscle was extracted with anhydrous diethyl ether, and the extract was dried to constant weight at 100 ± 5 °C.

### 2.6. Fatty Acid Composition Analysis

Fatty acid composition was analyzed as fatty acid methyl esters (FAMEs) by gas chromatography in accordance with the national standard GB 5009.168-2016. Briefly, an internal standard (triundecanoin) was added to homogenized thigh muscle samples, which were then hydrolyzed with 8.3 mol/L HCl at 70–80 °C. The liberated lipids were extracted with a diethyl ether-petroleum ether mixture (1:1, *v*/*v*). No antioxidants, such as butylated hydroxytoluene (BHT), were added during the FAME preparation procedure. The extracted fat was subjected to base-catalyzed transesterification using 2% sodium hydroxide in methanol, followed by reaction with 15% boron trifluoride-methanol. The resulting FAMEs were extracted with n-heptane and analyzed by GC-FID. The FAMEs were analyzed using an Agilent 7890A gas chromatograph equipped with a flame ionization detector (GC-FID; Agilent Technologies, Santa Clara, CA, USA) and separated on a DB-23 capillary column (60 m × 0.25 mm i.d., 0.15 μm film thickness; Agilent J&W, Agilent Technologies, Santa Clara, CA, USA). High-purity nitrogen was used as the carrier gas at a flow rate of 1 mL/min. The injection volume was 1 μL with a split ratio of 6:1, and the injector and detector temperatures were maintained at 280 °C. The oven temperature was programmed as follows: initially held at 90 °C for 2 min, increased to 180 °C at 10 °C/min and held for 1 min, then increased to 200 °C at 2 °C/min and held for 1 min, and finally increased to 230 °C at 3 °C/min and held for 2 min. The total run time was 35 min. Fatty acids were identified by comparison with a certified 37-component FAME standard mixture and quantified using the internal standard method.

### 2.7. Lipidomics Analysis

#### 2.7.1. Sample Preparation

Relative quantification of lipids in thigh muscle samples from AA, Chengkou, and Langshan chickens was performed using a 1290 Infinity high-performance liquid chromatography system (Agilent Technologies, Santa Clara, CA, USA) coupled with an Acquity HSS T3 column (2.1 mm × 100 mm, 1.8 μm; Waters, Milford, MA, USA) and a quadrupole time-of-flight mass spectrometer equipped with an electrospray ionization source (AB SCIEX 6600, Framingham, MA, USA). A total of 18 samples (6 biological replicates per breed) were randomly selected from the 30 individuals per breed and used for lipidomic analysis. For each sample, 40 mg of tissue was accurately weighed and transferred to a labeled tube. Subsequently, 300 μL of methanol containing deuterated internal standards was added for homogenization, followed by liquid-liquid extraction with 1 mL of methyl tert-butyl ether (MTBE). After mixing, 250 μL of water was added to induce phase separation. The mixture was centrifuged at 5000 rpm for 5 min at 4 °C, and 1 mL of the upper organic layer was collected. The organic phase was dried under a nitrogen stream and stored at −80 °C until analysis. For analysis, the dried sample was reconstituted in 200 μL of dichloromethane/methanol (2:1, *v*/*v*). For negative ion mode analysis, an aliquot of the reconstituted solution was transferred to an autosampler vial; for positive ion mode analysis, an additional 300 μL of dichloromethane/methanol (2:1, *v*/*v*) was added before injection.

#### 2.7.2. UPLC-QTOF-MS Conditions

Lipid separation was performed by gradient elution using acetonitrile/water (6:4, *v*/*v*) as mobile phase A and acetonitrile/isopropanol (1:9, *v*/*v*) as mobile phase B. The gradient program was set as follows: 0–1.0 min, 60% B; 1.0–15.0 min, linear increase from 60% to 100% B; 15.0–17.5 min, 100% B; 17.5–17.7 min, decrease to 60% B; and 17.7–20 min, 60% B. The total analysis time was 20 min at a flow rate of 300 μL/min. The autosampler and column temperatures were maintained at 10 °C and 50 °C, respectively, and the injection volume was 2 μL. Mass spectrometry parameters were set according to a previous report [16]. Data were acquired over the mass range of *m*/*z* 100–1200. Peak detection, alignment, and lipid identification were performed using MExplorer Ultimate software (V3.0.0.412; Dalian ChemDataSolution Information Technology Co., Ltd., Dalian, China). Lipids identified only by MS1 information or showing a relative standard deviation (RSD) higher than 20% in quality control (QC) samples were excluded. Relative quantification was based on the peak area of each lipid ion. The stability of the LC-MS system was monitored by inserting a QC sample after every 10 analytical runs. Overlays of the total ion current (TIC) chromatograms of QC samples in positive and negative ion modes are presented in Supplementary Figure S1.

### 2.8. Statistical Analysis

Statistical analyses were performed using IBM SPSS 25.0 (IBM Corp., Armonk, NY, USA). The normality of the data was assessed using the Shapiro–Wilk test, and homogeneity of variances was evaluated using Levene’s test before performing parametric analyses. One-way analysis of variance (ANOVA) followed by Tukey’s post-hoc test was applied to assess significant differences among the three breeds, with Tukey’s test used for multiple comparison adjustment. Differences at *p* < 0.05 were considered statistically significant. Data are expressed as mean ± standard deviation (SD). Multivariate statistical analyses, including principal component analysis (PCA) and partial least squares discriminant analysis (PLS-DA), were performed using MetaboAnalyst 5.0 to evaluate the overall distribution patterns and discrimination among different chicken breeds based on volatile compounds and lipidomic profiles. Histograms and graphical visualizations were constructed using GraphPad Prism 10.0 (GraphPad Software Inc., La Jolla, CA, USA).

## 3. Results

### 3.1. Effect of Breed on the Volatile Flavor Profile of Chicken Thigh Muscles

**Figure 1:**
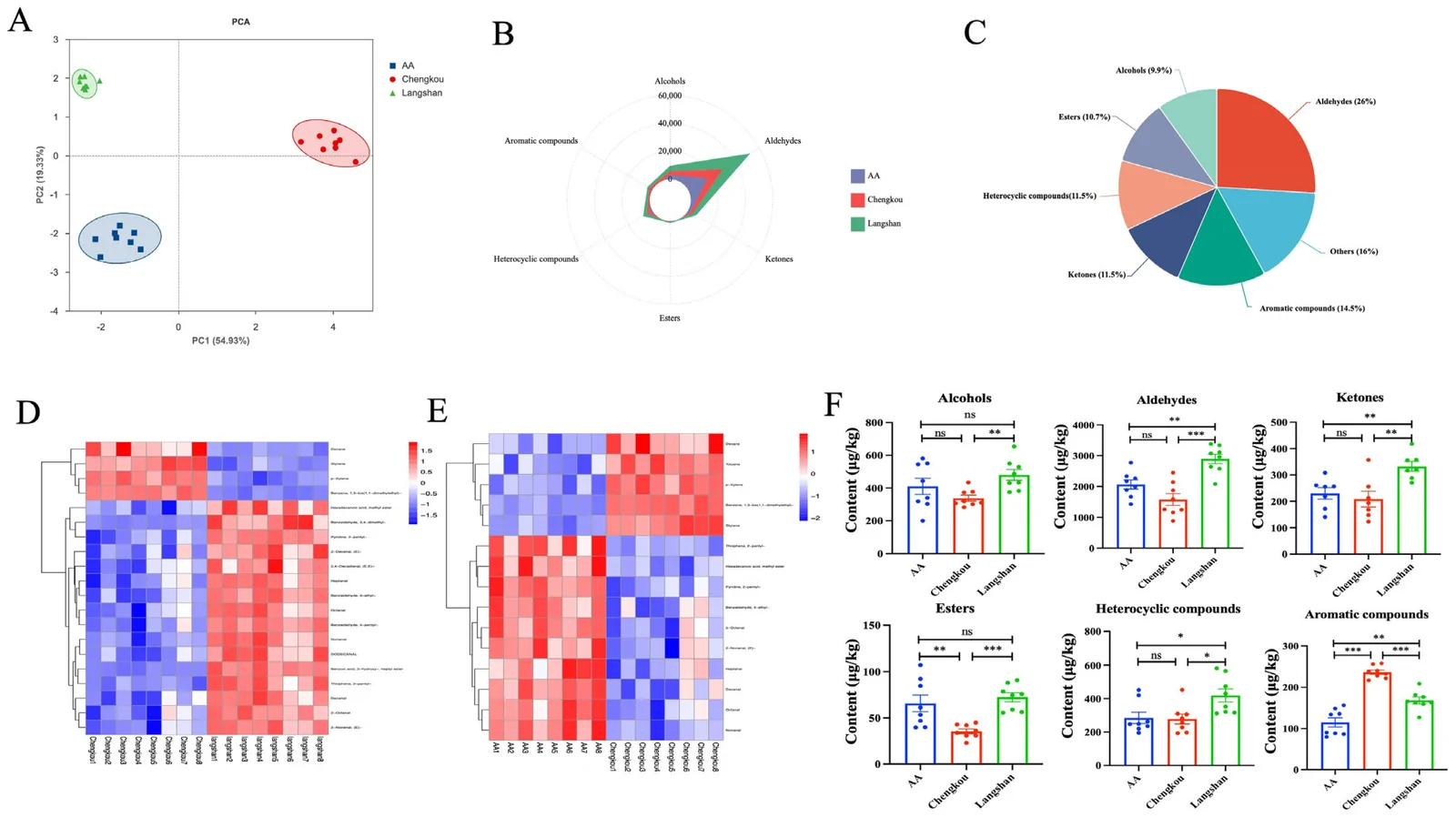
Volatile flavor profiles of thigh muscle from AA, Chengkou, and Langshan chickens. (**A**) PCA score plot of volatile compounds. (**B**) Radar plot showing the total content of volatile flavor compounds. (**C**) Pie chart showing the relative abundance of volatile compound classes. (**D**) Heatmap of differential volatile compounds between Langshan and Chengkou chickens. (**E**) Heatmap of differential volatile compounds between AA and Chengkou chickens. (**F**) Bar plot comparing the contents of main volatile compound classes among the three breeds. Data are presented as mean ± standard deviation (*n* = 8). * *p* < 0.05, ** *p* < 0.01, *** *p* < 0.001; ns, not significant.

To evaluate breed-dependent differences in volatile flavor profiles, the composition and contents of volatile flavor compounds in thigh muscle were analyzed in AA, Chengkou, and Langshan chickens. A total of 131 volatile compounds were identified and quantified. As shown in Figure 1A, principal component analysis (PCA) revealed that PC1 and PC2 explained 54.93% and 19.33% of the total variance, respectively, indicating clear separation among the three breeds. The corresponding PCA loading plot further showed the contributions of individual volatile compounds to the observed separation among the three breeds (Supplementary Figure S2). Aldehydes were the predominant class of volatile compounds in all breeds (Figure 1B,C). The total content of volatile flavor compounds was significantly higher in Langshan chickens than in the other two breeds (Figure 1B; *p* < 0.05). Furthermore, the contents of alcohols, aldehydes, ketones, esters, heterocyclic compounds, and aromatic compounds were also significantly higher in Langshan chickens than in AA and Chengkou chickens (Figure 1F; *p* < 0.05). In contrast, Chengkou chickens exhibited significantly higher contents of aromatic compounds than Langshan and Arbor Acres chickens (Figure 1F; *p* < 0.05). Based on the criteria of fold change (FC) > 1.5, *p* < 0.05, and variable importance in projection (VIP) > 1, 22 differential volatile compounds were identified. Clear differences in volatile profiles were observed between Langshan and Chengkou chickens (Figure 1D) as well as between AA and Chengkou chickens (Figure 1E). Notably, benzeneacetaldehyde was the only differential compound identified between AA and Langshan chickens; it was highly enriched in AA chickens, whereas it was present at lower levels in Langshan chickens.

### 3.2. Effect of Breed on the Amino Acid Composition of Chicken Thigh Muscles

Total amino acid (TAA) content differed significantly among the three breeds (Table 1), with Langshan chicken showing the highest content, followed by Chengkou chicken and Arbor Acres chicken (*p* < 0.05). Although statistically significant, the differences in TAA content among breeds were relatively small. Specifically, Asp, Gly, Val, Ile, and Tyr levels were significantly higher in both Chengkou and Langshan chickens than in AA chickens. Alanine (Ala) content was significantly higher in AA and Langshan chickens than in Chengkou chickens. Langshan chickens exhibited significantly higher levels of Thr, Ser, Glu, Met, Leu, Phe, and Arg compared with the other two breeds. In contrast, Lys and His contents were significantly higher in AA chickens than in Chengkou and Langshan chickens. No significant differences in Pro content were observed among the three breeds.

### 3.3. Effect of Breed on the Fat Content and Fatty Acid Composition of Chicken Thigh Muscles

**Figure 2:**
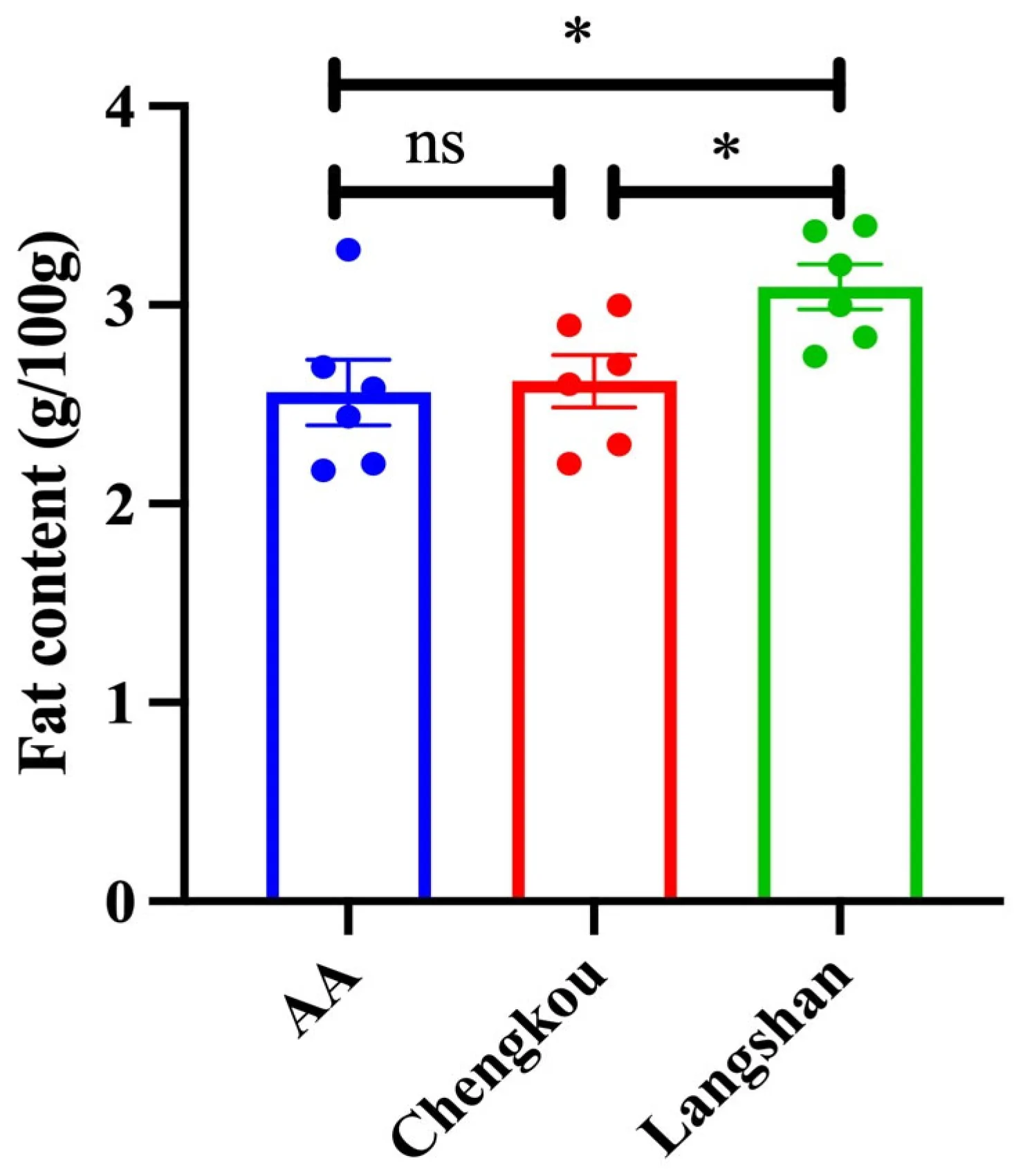
Breed-dependent differences in thigh muscle fat content from AA, Chengkou, and Langshan chickens. Fat content (g/100 g) in thigh muscle of Arbor Acres (AA), Chengkou, and Langshan chickens. Data are presented as mean ± standard deviation (*n* = 6). * *p* < 0.05; ns, not significant.

The total fat content and fatty acid composition of thigh muscles are presented in Figure 2 and Table 2. As shown in Figure 2, the total fat content of Langshan chickens was significantly higher than that of AA and Chengkou chickens (*p* < 0.05), while no significant difference was observed between Chengkou and AA chickens. A total of seventeen fatty acids were identified (Table 2), including five saturated fatty acids (SFAs), five monounsaturated fatty acids (MUFAs), and seven polyunsaturated fatty acids (PUFAs). C22:5n3 (DPA) was not detected in any of the three breeds. Among these fatty acids, oleic acid (C18:1n9c), linoleic acid (C18:2n6c), palmitic acid (C16:0), and stearic acid (C18:0) were the predominant fatty acids detected in the thigh muscles of all three chicken breeds. No significant differences were observed in the total levels of SFAs and MUFAs among the three breeds. The contents of α-linolenic acid (C18:3n3) and eicosadienoic acid (C20:2n6) were significantly higher in AA chickens than in Chengkou and Langshan chickens. Regarding arachidonic acid (C20:4n6), both Langshan and Chengkou chickens exhibited significantly higher levels than AA chickens (*p* < 0.05), with no significant difference between the two indigenous breeds. In contrast, docosahexaenoic acid (DHA, C22:6n3) was significantly higher only in Chengkou chickens compared with both AA and Langshan chickens (*p* < 0.05). The calculated fatty acid nutritional indices further indicated differences in the overall fatty acid profiles among the three breeds. Chengkou chickens showed the highest PUFA/SFA ratio, HH, and HPI values and the lowest IA and IT values, whereas Langshan chickens showed the highest *n*-6/*n*-3 PUFA ratio (Supplementary Table S3). Additionally, the total fatty acid methyl esters (total FAME) were numerically higher in Chengkou chickens than in AA and Langshan chickens, although the differences were not statistically significant (Table 2).

### 3.4. Effect of Breed on the Lipid Composition of Chicken Thigh Muscles

**Figure 3:**
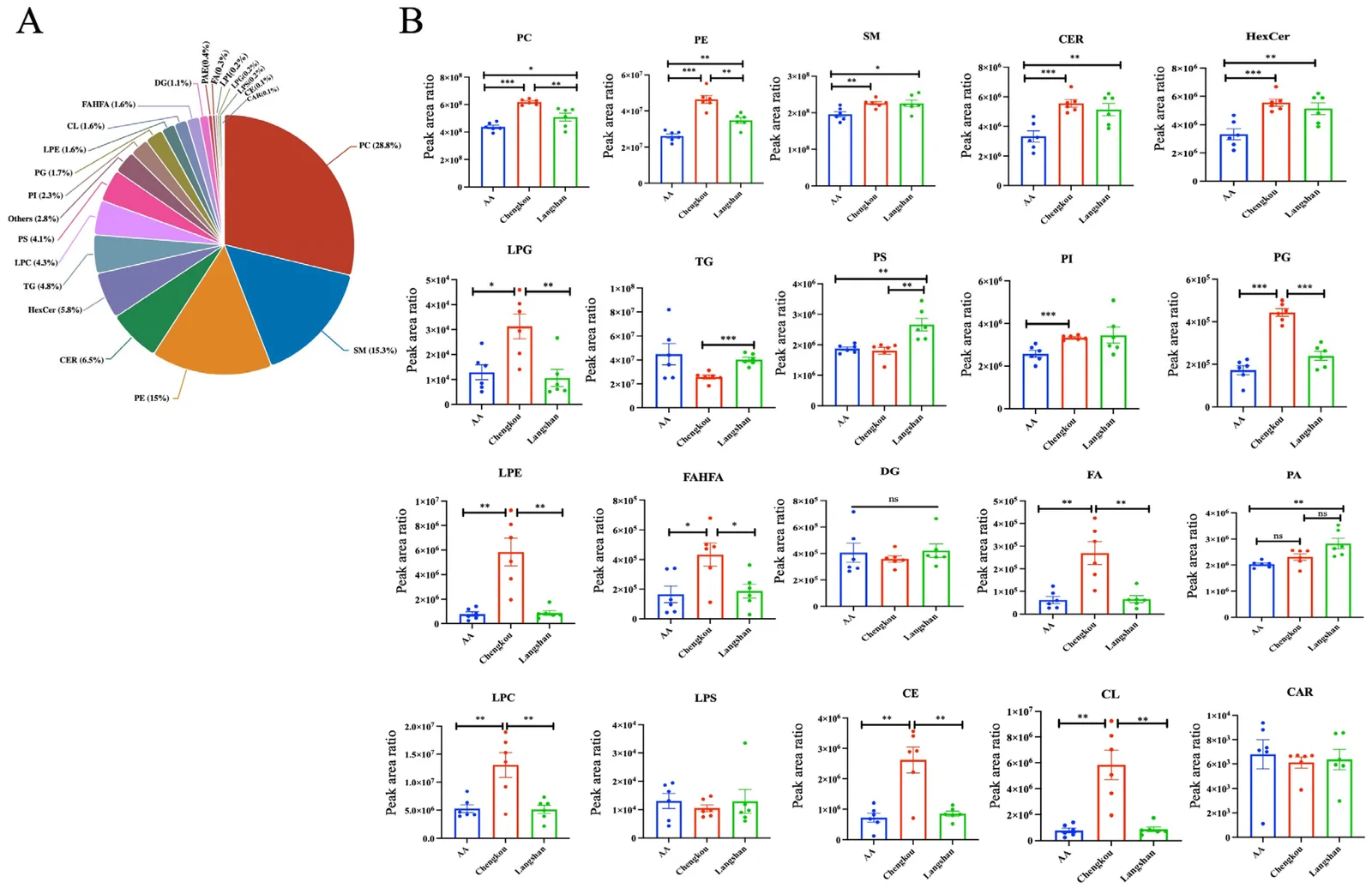
Lipidomic profiles and breed-dependent differences in thigh muscle from AA, Chengkou, and Langshan chickens. (**A**) Pie chart showing the distribution of the 787 lipid species identified. (**B**) Bar chart with scatter plots showing the relative abundance of major lipid subclasses in the three breeds. Data are presented as mean ± standard deviation (*n* = 6). * *p* < 0.05, ** *p* < 0.01, *** *p* < 0.001; ns, not significant.

The lipid profiles of chicken thigh muscle samples from AA, Chengkou, and Langshan chickens were characterized using UPLC-ESI-MS/MS. A total of 787 lipid species were identified and classified into six major classes: glycerophospholipids (GP, 63.02%), sphingolipids (SP, 26.05%), glycerolipids (GL, 5.72%), fatty acyls (FA, 2.03%), sterol lipids (ST, 0.13%), and unclassified lipids (2.80%). Glycerophospholipids and sphingolipids were the predominant classes. The subclass distribution is shown in Figure 3A, with phosphatidylcholine (PC, 28.8%), sphingomyelin (SM, 15.3%), and phosphatidylethanolamine (PE, 15.0%). Further classification revealed two subclasses of glycerolipids (DG and TG), twelve subclasses of glycerophospholipids (CL, LPC, LPE, LPG, LPI, LPS, PA, PC, PE, PG, PI, and PS), three subclasses of sphingolipids (Cer, HexCer, and SM), two subclasses of fatty acyls (FA and FAHFA), and one subclass of sterol lipids (CE). Significant differences in the abundance of specific lipid subclasses were observed among the three breeds (Figure 3B). Within glycerophospholipids, Chengkou chickens exhibited significantly higher levels of PC, PG, LPC, LPE, LPG, and CL than both AA and Langshan chickens (*p* < 0.001). PA was significantly more abundant in Langshan chickens than in AA and Chengkou chickens (*p* < 0.001). PS was significantly higher in Langshan and Chengkou chickens than in AA chickens (*p* < 0.001), while PI was significantly more abundant in Chengkou chickens than in AA chickens (*p* < 0.001). Regarding sphingolipids, Chengkou and Langshan chickens showed significantly higher SM, Cer, and HexCer levels than AA chickens (*p* < 0.05). For glycerolipids, TG content was significantly higher in Langshan chickens than in Chengkou chickens (*p* < 0.001). Additionally, FA, FAHFA, and CE were significantly more abundant in Chengkou chickens than in the other two breeds (*p* < 0.05).

### 3.5. Comparative Lipidomic Analysis of Chicken Thigh Muscles from Different Breeds

**Figure 4:**
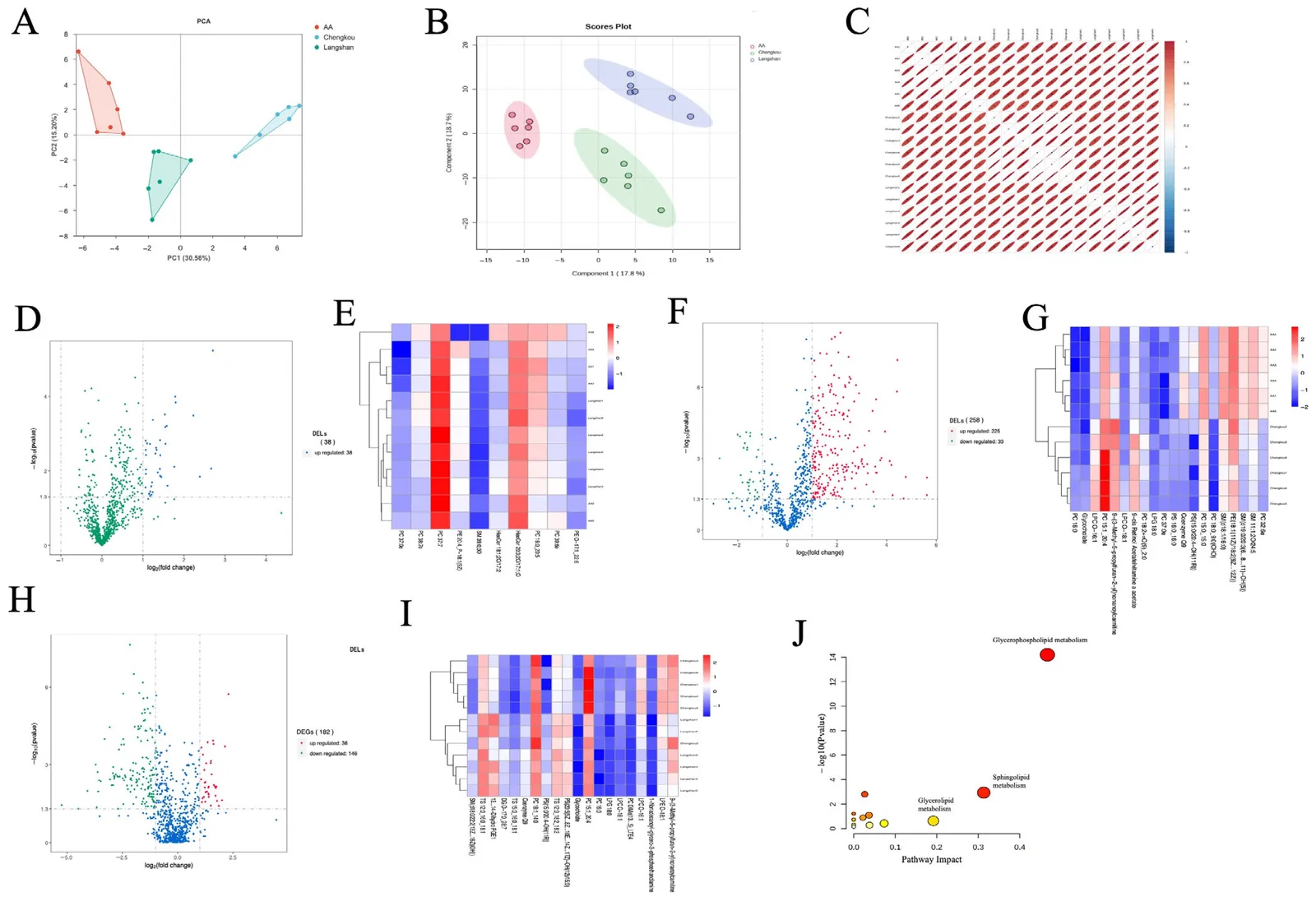
Integrated lipidomic profiling of thigh muscles from different chicken breeds. (**A**) Principal component analysis (PCA) score plot. (**B**) Partial least squares discriminant analysis (PLS-DA) score plot. (**C**) Pearson correlation analysis of all samples. (**D**) Volcano plot of differentially abundant lipids between AA and Langshan chickens (blue: upregulated; green: not significant). (**E**) Heatmap of differential lipids between AA and Langshan breeds (top 10 upregulated). (**F**) Volcano plot of differentially abundant lipids between AA and Chengkou chickens (red: upregulated; green: downregulated). (**G**) Heatmap of differential lipids between AA and Chengkou breeds (top 10 upregulated and top 10 downregulated). (**H**) Volcano plot of differentially abundant lipids between Chengkou and Langshan chickens (red: upregulated; green: downregulated). (**I**) Heatmap of differential lipids between Chengkou and Langshan breeds (top 10 upregulated and top 10 downregulated). (**J**) KEGG pathway enrichment analysis, where bubble size represents the number of enriched differential lipids and color indicates the significance level (*p*-value). Only pathways with *p* < 0.05 are displayed. Data are presented as mean ± standard deviation (*n* = 6).

To investigate breed-specific differences in lipid metabolism among AA, Chengkou, and Langshan chickens, untargeted lipidomics was performed. PCA and partial least squares discriminant analysis (PLS-DA) score plots showed differences in lipidomic profiles among the three breeds (Figure 4A,B), and the correlation heatmap confirmed high intra-group similarity (Figure 4C), indicating distinct lipid profiles in thigh muscles. The corresponding PCA and PLS-DA loading plots further showed the lipid variables contributing to the observed separation among the three breeds (Supplementary Figure S3A,B). Between AA and Langshan chickens, 38 differentially abundant lipids (DLs) were identified, all of which were upregulated in Langshan chickens compared with AA chickens (Figure 4D). The heatmap of the top 10 upregulated DLs (Figure 4E) showed clear clustering of Langshan samples. Between AA and Chengkou chickens, 258 DLs were detected, including 225 upregulated and 33 downregulated lipids in Chengkou chickens (Figure 4F). The heatmap of the top 10 upregulated and top 10 downregulated DLs (Figure 4G) highlighted the most altered lipids between the two breeds. Between Chengkou and Langshan chickens, 182 DLs were identified, with 36 upregulated and 146 downregulated lipids in Langshan chickens (Figure 4H). The corresponding heatmap (Figure 4I) revealed distinct lipid signatures between the two indigenous breeds. To gain insight into the biological functions of these DLs, KEGG pathway enrichment analysis was performed. As shown in Figure 4J and Supplementary Table S2, the DLs were mainly enriched in glycerolipid metabolism, sphingolipid metabolism, and glycerophospholipid metabolism (*p* < 0.05).

### 3.6. Correlation Analysis of Volatile Compounds, Fatty Acids, and Differential Lipid Profiles of Chicken Thigh Muscles from Different Breeds

**Figure 5:**
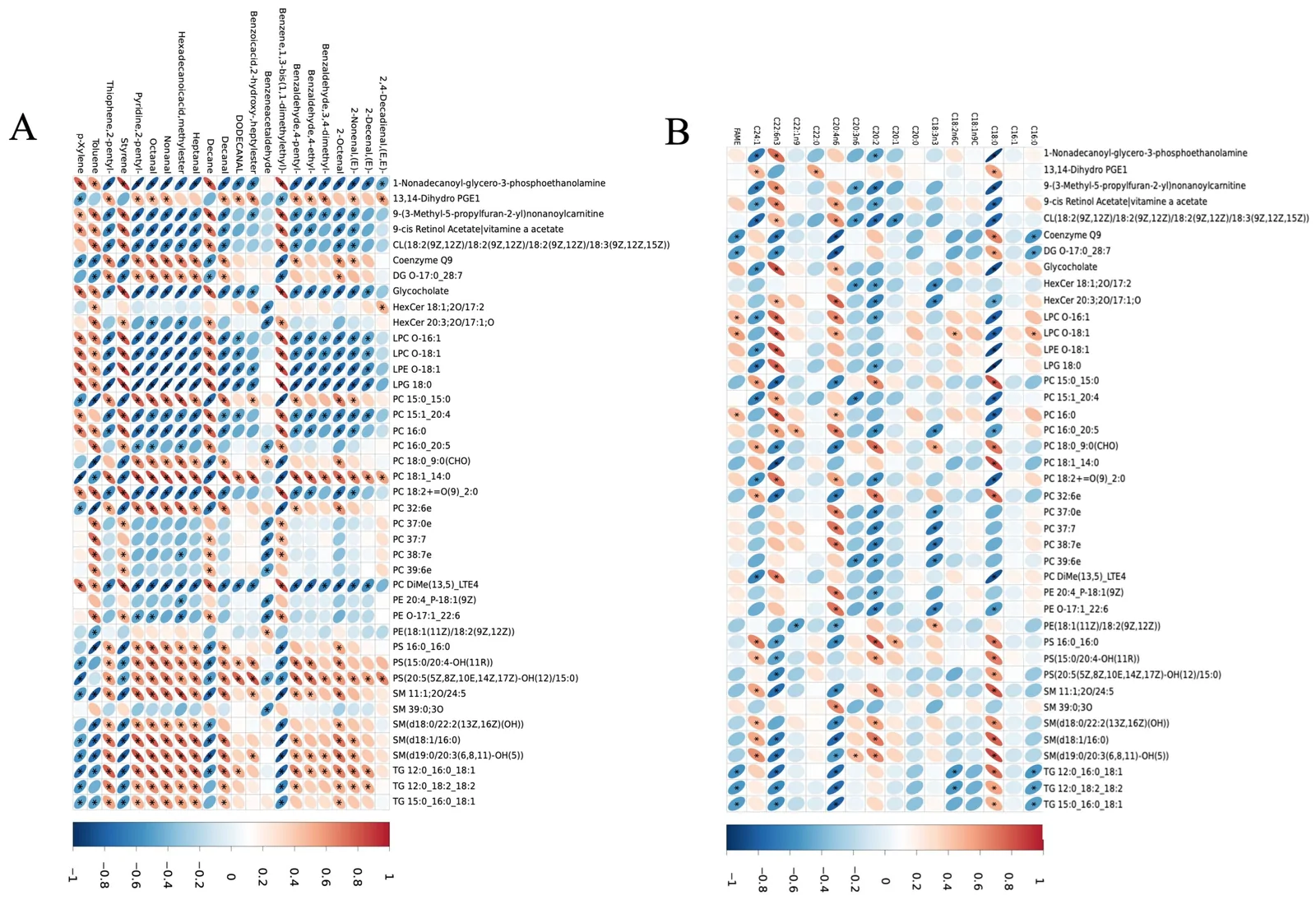
Pearson correlation heatmaps. (**A**) Correlation between volatile compounds and lipid subclasses; (**B**) correlation between fatty acids and lipid subclasses. Red indicates positive correlation; blue indicates negative correlation. * *p* < 0.05.

Lipid biomarkers may provide insights into the flavor-related and nutritional characteristics of chicken meat. To explore the associations between differential lipids and flavor-related and nutritional characteristics in different chicken breeds, Pearson correlation coefficients were determined. Pearson correlation analysis was performed to investigate the associations between differential lipid biomarkers and volatile compounds and fatty acids. For volatile compounds (Figure 5A), nonanal, 1,3-di-tert-butylbenzene, and styrene showed extremely strong and significant correlations with lysophosphatidylglycerol LPG 18:0 (*r* > 0.88, *p* < 0.001), suggesting that glycerophospholipid-related lipids may be associated with the formation of lipid-derived volatile flavor compounds in chicken meat. For fatty acids (Figure 5B), stearic acid (C18:0) showed the strongest negative correlation with LPG 18:0 (*r* = −0.9305, *p* < 0.001), and polyunsaturated fatty acids (C20:2, C22:6n3, C20:4n6) were significantly correlated with glycerophospholipid species (*r* > 0.70, *p* < 0.01), suggesting an association between fatty acid composition and lipid remodeling in chicken thigh muscle.

## 4. Discussion

In this study, we observed marked breed-associated differences in flavor-related compounds, nutritional characteristics, fatty acid composition, and lipidomic profiles of thigh muscle. Indigenous Langshan and Chengkou chickens exhibited distinct meat quality attributes compared with commercial AA broilers, which were associated with differences in flavor-related metabolites and lipid metabolism-related characteristics. These findings highlight the potential role of breed-associated metabolic differences in shaping the nutritional and flavor-related properties of chicken meat.

Chemical composition serves as the foundation of food aroma. With the continuous advancement of analytical techniques and instrumentation, an increasing number of aroma compounds and their contributions have been identified. Aroma profiles are inherently complex, typically comprising alcohols, esters, organic acids, ketones, aldehydes, furans, pyrazines, hydrocarbons, and sulfur-containing compounds [19,20], which exhibit wide variations in polarity, solubility, volatility, and thermal stability [15]. It should be noted that the volatile compounds measured in raw meat represent the potential flavor profile of raw meat rather than the final cooked flavor, because thermal processing (e.g., cooking) generates additional aroma compounds through reactions such as the Maillard reaction and Strecker degradation [21,22]. As shown in Figure 1A, principal component analysis (PCA) revealed distinct distribution patterns among the three breeds based on their volatile profiles, indicating marked breed-dependent differences in the volatile composition of chicken thigh muscle. Aldehydes were the most abundant class of volatile compounds across all breeds, consistent with their established role as key contributors to chicken meat aroma [17,23]. Moreover, the higher total volatile content observed in Langshan chickens suggests that indigenous black-feathered breeds, particularly Langshan, may possess distinct volatile profiles compared with commercial broilers. The differences in volatile profiles among breeds may reflect variations in the abundance of flavor precursors and metabolic characteristics associated with breed-specific muscle composition. The breed-associated variation in benzeneacetaldehyde suggests that individual aldehyde compounds may be associated with differences in aroma characteristics among chicken breeds [17].

Amino acids are critical determinants of meat nutritional value and flavor, serving as both protein constituents and precursors for taste development [24,25]. In the present study, significant breed-dependent differences in amino acid composition were observed. Langshan chickens exhibited the highest total amino acid (TAA) content, followed by Chengkou chickens, while AA broilers had the lowest TAA content, indicating that indigenous black-feathered breeds possess distinct nutritional characteristics. Notably, the umami-associated amino acids Asp and Glu, as well as several sweet-taste amino acids (Thr, Ser, Gly), showed breed-specific distribution patterns. Specifically, Asp, Glu, Thr, Ser, Gly, and Val were significantly higher in Langshan or Chengkou chickens than in AA chickens, whereas Ala was higher in AA and Langshan chickens than in Chengkou chickens. Among these, Glu—a key contributor to umami was particularly elevated in Langshan chickens, consistent with their higher total volatile content. Amino acids such as Glu are known to participate in flavor formation via the Maillard reaction and Strecker degradation during thermal processing [17]. Additionally, the bitter-taste amino acid Tyr was also significantly higher in both indigenous breeds. Furthermore, the contents of most essential amino acids (Thr, Val, Met, Ile, Leu, Phe, Arg) were significantly higher in Langshan and Chengkou chickens than in the AA breed, further supporting the distinct nutritional characteristics of indigenous breeds compared with fast-growing commercial lines [26]. In contrast, AA chickens showed significantly higher levels of Lys and His, suggesting breed-specific differences in amino acid metabolism. Collectively, these findings indicate that breed variation influences both the nutritional composition and flavor-related amino acid profiles of chicken thigh muscle, highlighting the potential contribution of indigenous breeds to diverse meat quality characteristics.

Chicken meat is highly appreciated by consumers due to its sensory attributes and nutritional value, such as a high content of unsaturated fatty acids and low levels of fat, sodium, and cholesterol [12]. Fatty acids are primarily derived from the hydrolysis of triglycerides and phospholipids [27], and the presence of unsaturated fatty acids (UFAs) in meat is considered beneficial to human health [28]. Total fat content is a key determinant of meat juiciness, tenderness, and flavor [25]. In this study, the total fat content of Langshan chickens was significantly higher than that of AA and Chengkou chickens, while no significant difference was observed between Chengkou and AA chickens. This finding is consistent with recent studies showing that local chicken breeds often have distinct fatty acid profiles compared with fast-growing commercial broilers [5,6]. Notably, both Langshan and Chengkou chickens exhibited significantly higher levels of C20:4n6 than AA chickens, with no significant difference between the two indigenous breeds. In contrast, C22:6n3 was significantly higher only in Chengkou chickens compared with both AA and Langshan chickens. These results suggest that C20:4n6 may represent a characteristic fatty acid associated with the two indigenous black-feathered breeds examined in this study, whereas the higher C22:6n3 content in Chengkou chickens indicates breed-associated variation in DHA abundance. It has been reported that arachidonic acid (ARA) and DHA-containing glycerophospholipids are key differential molecules between chicken breeds [10]. Our results highlight the breed-associated differences in fatty acid composition, consistent with the notion that indigenous breeds often exhibit distinct lipid profiles compared to commercial lines. In contrast, AA chickens exhibited higher levels of C18:3n3 and C20:2n6, suggesting breed-associated differences in fatty acid composition. Additionally, the higher PUFA content in Chengkou chickens may be associated with differences in flavor-related volatile compounds, as PUFAs can serve as potential precursors for some volatile compounds generated during lipid oxidation pathways [23]. However, lipid oxidation is a complex process, and excessive oxidation may also lead to undesirable flavor formation and reduced meat quality. Therefore, the relationship between PUFA abundance and flavor characteristics should be interpreted cautiously, as lipid oxidation can generate both desirable aroma compounds and undesirable oxidative products depending on oxidation extent and processing conditions. Overall, indigenous black-feathered breeds, particularly Chengkou, exhibited distinct fatty acid profiles that may be associated with differences in nutritional characteristics and flavor-related properties of chicken meat [25,29]. The calculated fatty acid nutritional indices provided an additional perspective on these breed-associated differences (Supplementary Table S3). Chengkou chickens exhibited the highest PUFA/SFA ratio, HH, and HPI values and the lowest IA and IT values among the three breeds, whereas Langshan chickens showed the highest *n*-6/*n*-3 PUFA ratio. These indices provide an integrated assessment of fatty acid composition; however, their nutritional implications should be interpreted cautiously because the present study was designed primarily to characterize breed-associated differences rather than to evaluate clinical or dietary health outcomes.

Recent lipidomic studies have demonstrated that glycerophospholipids, sphingolipids, and glycerolipids undergo marked changes during animal growth and are closely associated with meat quality traits in local chicken breeds [10,30]. Therefore, lipidomic profiling provides an important approach for investigating whether breed-associated differences in lipid metabolism contribute to variations in nutritional and flavor-related characteristics of chicken meat. In the present study, UPLC-ESI-MS/MS-based lipidomic analysis of thigh muscle identified 787 lipids, which were mainly classified as glycerophospholipids (63.02%), sphingolipids (26.05%), and glycerolipids (5.72%). Multivariate analysis (PCA and PLS-DA) revealed distinct distribution patterns among the three breeds, indicating distinct lipid profiles. The extensive differences in lipid profiles among breeds indicate that indigenous and commercial chickens display breed-associated lipid metabolic remodeling patterns, rather than differences limited to individual lipid molecules. Although we did not quantitatively classify these DLs into subclasses, KEGG enrichment analysis showed that they were mainly enriched in glycerolipid metabolism, sphingolipid metabolism, and glycerophospholipid metabolism. These enriched pathways suggest that breed-associated variation in lipid metabolism may contribute to the formation of distinct lipid compositions and may be related to differences in meat quality characteristics among chicken breeds. This finding is consistent with the observation that glycerophospholipids and sphingolipids are the predominant lipid classes in the total lipidome, and similar enrichment patterns have been reported in comparative lipidomic studies of indigenous and commercial chickens [31]. Therefore, the breed-associated differences in these pathways may reflect distinct metabolic patterns among commercial and indigenous chickens, which may further contribute to variations in lipid composition and related meat quality traits.

To further interpret the potential biological significance of these pathways, we examined the biochemical roles of these pathways. Glycerophospholipids serve as reservoirs for polyunsaturated fatty acids (PUFAs), which can undergo autoxidation to generate volatile aldehydes, ketones, and alcohols [23]. It has been shown that glycerophospholipids (GPs) are highly positively correlated with unsaturated fatty acids and may be associated with the formation of meat flavor aldehydes [30]. Sphingolipids, including sphingomyelin (SM) and ceramides (Cer), are key components of membrane microdomains and may influence the accumulation of flavor-related metabolites [32]. Glycerolipid metabolism reflects fatty acid turnover and may be associated with the availability of free fatty acids for subsequent oxidation into volatiles during cooking [17]. Correlation analysis further revealed associations between differential lipids and flavor-related compounds. We observed strong positive correlations of LPG 18:0 with three volatile compounds (nonanal, 1,3-di-tert-butylbenzene, styrene), suggesting that glycerophospholipid metabolism-related changes may be associated with differences in flavor-related volatile compound profiles. Stearic acid (C18:0) showed a strong negative correlation with LPG 18:0, suggesting an association between fatty acid composition and lysophospholipid-related changes. In addition, polyunsaturated fatty acids (C20:2n6, C22:6n3, C20:4n6) were significantly correlated with glycerophospholipids. However, these correlation relationships should be interpreted cautiously, as Pearson correlation analysis indicates statistical associations but does not establish causal relationships or regulatory mechanisms. Further functional validation, including enzyme activity assays, gene expression analysis, and metabolic flux studies, is required to clarify the biological mechanisms underlying these associations.

Although the calculated fatty acid nutritional indices provide an integrated perspective on the fatty acid profile, they do not directly establish the dietary or clinical health effects of chicken meat. Therefore, future studies incorporating consumer-oriented nutritional evaluations and comparisons with other dietary protein sources are needed to further characterize the dietary value of chicken lipids. Furthermore, although the present study revealed breed-associated differences in fatty acid composition and lipidomic profiles, differences in dietary composition under different production conditions may also have contributed to the observed variations. In particular, lard was used as the primary dietary fat source, and its fatty acid composition may have influenced the fatty acid composition of thigh muscles to some extent. Since the three breeds were raised according to their respective commercial production practices and harvested at different market ages, the independent effects of breed and diet could not be completely distinguished under the current experimental design. Future studies using standardized diets across breeds are warranted to further clarify the contribution of breed-associated factors and dietary factors to chicken lipid characteristics.

The volatile and lipidomic analyses in the present study were exploratory in nature. Because the amino acid, fatty acid, volatile compound, and lipidomic datasets represent distinct analytical domains, they were not treated as a single study-wide hypothesis family. For the exploratory volatile and lipidomic analyses, differential features were therefore screened using nominal *p*-values together with additional feature-selection criteria rather than applying a single study-wide FDR correction across all analytical datasets. Although this strategy may retain potentially informative features in a study with relatively limited biological replication, it may also increase the possibility of false-positive findings. Therefore, the identified volatile compounds and differential lipids should be regarded as exploratory candidates, and further validation in independent populations is warranted.

Overall, this integrated multi-omics approach provides new insights into the biological basis of breed-associated differences in chicken meat quality by linking lipid metabolic characteristics with flavor-related compounds and nutritional traits. These findings provide a foundation for future studies aimed at further elucidating how genetic background and metabolic characteristics contribute to differences in poultry meat quality.

## 5. Conclusions

The present study reveals that indigenous black-feathered chickens (Langshan and Chengkou) exhibit distinct nutritional and flavor-related characteristics compared with commercial white-feathered AA broilers, particularly in volatile flavor compounds, amino acid composition, fatty acid profiles, and lipid profiles. By integrating multi-omics approaches, this study provides new insights into breed-associated differences in chicken meat quality and highlights the potential role of lipid metabolism in shaping these differences. Correlation analysis identified potential lipid molecules associated with breed-related quality traits, including phosphatidic acid (PA) in Langshan chickens, higher DHA abundance in Chengkou chickens, and C20:4n6 as a characteristic fatty acid of black-feathered breeds. Future studies involving targeted validation and functional analyses are needed to further elucidate the mechanisms underlying breed-associated differences in chicken meat quality.

## Figures and Tables

**Table 1:** The contents of amino acids in chicken among different breeds groups.

		Contents (g/100 g Meat Sample)		
Amino Acids	Taste Attributes	AA Chicken	Chengkou Chicken	Langshan Chicken
Asp	Umami	1.62 ± 0.04 ^b^	1.68 ± 0.05 ^a^	1.68 ± 0.03 ^a^
Thr *	Sweet	1.02 ± 0.02 ^b^	1.04 ± 0.03 ^b^	1.13 ± 0.03 ^a^
Ser	Sweet	1.13 ± 0.03 ^b^	1.14 ± 0.03 ^b^	1.22 ± 0.04 ^a^
Glu	Umami	2.21 ± 0.06 ^c^	2.35 ± 0.08 ^b^	2.81 ± 0.06 ^a^
Gly	Sweet	0.72 ± 0.05 ^b^	0.81 ± 0.05 ^a^	0.84 ± 0.10 ^a^
Ala	Sweet	1.25 ± 0.05 ^a^	1.19 ± 0.05 ^b^	1.27 ± 0.07 ^a^
Val *	Sweet	0.34 ± 0.06 ^b^	0.42 ± 0.02 ^a^	0.46 ± 0.02 ^a^
Met *	Bitter	0.99 ± 0.06 ^c^	1.06 ± 0.03 ^b^	1.15 ± 0.02 ^a^
Ile *	Bitter	1.30 ± 0.03 ^b^	1.71 ± 0.05 ^a^	1.75 ± 0.03 ^a^
Leu *	Bitter	0.49 ± 0.01 ^c^	0.53 ± 0.02 ^b^	0.56 ± 0.02 ^a^
Tyr	Bitter	0.24 ± 0.03 ^b^	0.99 ± 0.06 ^a^	1.00 ± 0.02 ^a^
Phe *	Bitter	0.72 ± 0.02 ^c^	1.96 ± 0.05 ^b^	2.09 ± 0.06 ^a^
Lys *	Sweet	1.35 ± 0.05 ^a^	0.13 ± 0.01 ^b^	0.14 ± 0.02 ^b^
His	Bitter	0.89 ± 0.28 ^a^	0.66 ± 0.04 ^b^	0.66 ± 0.03 ^b^
Arg	Bitter	0.96 ± 0.03 ^c^	1.12 ± 0.03 ^b^	1.20 ± 0.03 ^a^
Pro	Sweet	0.75 ± 0.04	0.83 ± 0.08	0.78 ± 0.05
TAA		15.99 ± 0.34 ^c^	17.63 ± 0.54 ^b^	18.74 ± 0.51 ^a^

Note: Values are presented as mean ± standard deviation (*n* = 6). * represents essential amino acids. Taste attributes of amino acids were assigned according to previous reports [17,18]. ^a,b,c^ Different superscripts within the same row indicate significant differences (*p* < 0.05). The same is below. Asp, aspartic acid; Thr, threonine; Ser, serine; Glu, glutamic acid; Gly, glycine; Ala, alanine; Val, valine; Met, methionine; Ile, isoleucine; Leu, leucine; Tyr, tyrosine; Phe, phenylalanine; Lys, lysine; His, histidine; Arg, arginine; Pro, proline; Cys, cystine; TAA, total amino acids.

**Table 2:** The contents of fatty acids in chicken among different breed groups.

Fatty Acid Contents (g/100 g Thigh Muscle, Wet Weight)	AA Chicken	Chengkou Chicken	Langshan Chicken
Palmitic acid (C16:0)	0.3050 ± 0.0357	0.3901 ± 0.1121	0.3273 ± 0.1335
Palmitoleic aci(C16:1n7)	0.046 ± 0.010	0.057 ± 0.030	0.055 ± 0.026
Stearic acid (C18:0)	0.2294 ± 0.0177	0.2699 ± 0.0720	0.2435 ± 0.1081
Oleic acid (C18:1n9c)	0.3526 ± 0.0379	0.4134 ± 0.1250	0.3859 ± 0.1667
Linoleic acid (C18:2n6c)	0.3323 ± 0.0755	0.4051 ± 0.1244	0.2673 ± 0.1160
α-Linolenic acid (C18:3n3)	0.0154 ± 0.0019 ^a^	0.0098 ± 0.0035 ^b^	0.0079 ± 0.0034 ^b^
Arachidic acid (C20:0)	0.0046 ± 0.0009	0.0055 ± 0.0014	0.0051 ± 0.0019
Eicosenoic acid (C20:1n9)	0.0055 ± 0.0005	0.0046 ± 0.0013	0.0050 ± 0.0025
Eicosadienoic acid (C20:2n6)	0.0144 ± 0.0012 ^a^	0.0070 ± 0.0020 ^b^	0.0083 ± 0.0044 ^b^
Eicosatrienoic acid (C20:3n6)	0.0262 ± 0.0028	0.0210 ± 0.0074	0.0224 ± 0.0084
Arachidonic acid (C20:4n6)	0.0688 ± 0.0615 ^b^	0.2416 ± 0.0519 ^a^	0.1715 ± 0.0810 ^a^
Behenic acid (C22:0)	0.0049 ± 0.0005	0.0045 ± 0.0012	0.0065 ± 0.0029
Eicosapentaenoic acid (EPA, C20:5n3)	0.0028 ± 0.0001	0.0025 ± 0.0010	nd
Erucic acid (C22:1n9)	0.0027 ± 0.0018	0.0027 ± 0.0008	0.0030 ± 0.0015
Lignoceric acid (C24:0)	0.0031 ± 0.0006	nd	0.0059 ± 0.0026
Nervonic acid (C24:1 n9)	0.0185 ± 0.0023	0.0097 ± 0.0028	0.0174 ± 0.0114
Docosahexaenoic acid (DHA, C22:6n3)	0.0117 ± 0.0027 ^b^	0.0360 ± 0.0116 ^a^	0.0117 ± 0.0056 ^b^
Total FAME (g/100 g)	1.4434 ± 0.1964	1.8885 ± 0.5273	1.5437 ± 0.6567

Note: Fatty acid contents are expressed as g/100 g thigh muscle on a wet-weight basis. Values are presented as mean ± SD (*n* = 6). Different superscript letters within the same row indicate significant differences among breeds (*p* < 0.05). nd, not detected; EPA, eicosapentaenoic acid; DHA, docosahexaenoic acid; Total FAME, total fatty acid methyl esters.

## Data Availability

The original data presented in this study are available upon reasonable request from the corresponding author.

## Supplementary Materials

The following supporting information can be downloaded at: https://www.mdpi.com/article/10.3390/foods15173098/s1, Table S1: Feed Formulation and Nutritional Levels of Experimental Chickens; Table S2: Full list of KEGG pathway enrichment results for differential lipids in chicken thigh muscle; Table S3: Fatty acid nutritional indices of thigh muscle from different chicken breeds; Figure S1: TIC overlap of QC samples for lipidomics in negative (A) and positive (B) ion modes; Figure S2: PCA loading plot of volatile compounds in thigh muscle from AA, Chengkou, and Langshan chickens. The numbered points indicate the top 10 volatile variables contributing most strongly to PC1 and PC2; Figure S3: Loading plots of the lipidomic multivariate analyses. (A) PCA loading plot showing the lipid variables contributing to the separation among AA, Chengkou, and Langshan chickens. (B) PLS-DA loading plot showing the lipid variables contributing to discrimination among the three breeds. The numbered points indicate the top 10 contributing variables; Figure S4: Permutation test validation of PLS-DA models based on lipidomic profiles. (A) AA broilers versus Chengkou chickens; (B) AA broilers versus Langshan chickens; (C) Chengkou chickens versus Langshan chickens. A total of 200 permutations were performed to evaluate model robustness and potential overfitting; Figure S5: Correlation between amino acids and lipid subclasses.
