# Supplementary material for: Comparative Analysis of Volatile Compounds, Amino Acids, Fatty Acids, and Lipidomic Profiles in Thigh Muscles of Commercial Arbor Acres (AA) Broilers and Indigenous Chengkou and Langshan Chickens
Source: Foods. 2026 Aug 31;15(17):3098. doi: 10.3390/foods15173098 (PMC13565686; doi:10.3390/foods15173098)
Supplement: Supplementary file 1 [file foods-15-03098-s001.zip › Supplementary Figure S2.pdf]

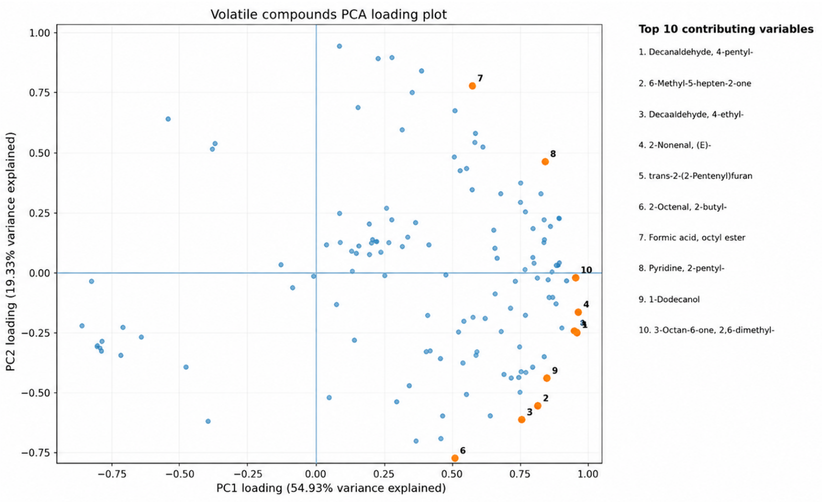

Supplementary Figure S2. PCA loading plot of volatile compounds in thigh muscle from AA, Chengkou, and Langshan chickens. The numbered points indicate the top 10 volatile variables contributing most strongly to PC1 and PC2.
