# Supplementary material for: Comparative Analysis of Volatile Compounds, Amino Acids, Fatty Acids, and Lipidomic Profiles in Thigh Muscles of Commercial Arbor Acres (AA) Broilers and Indigenous Chengkou and Langshan Chickens
Source: Foods. 2026 Aug 31;15(17):3098. doi: 10.3390/foods15173098 (PMC13565686; doi:10.3390/foods15173098)
Supplement: Supplementary file 1 [file foods-15-03098-s001.zip › Supplementary Figure S3.pdf]

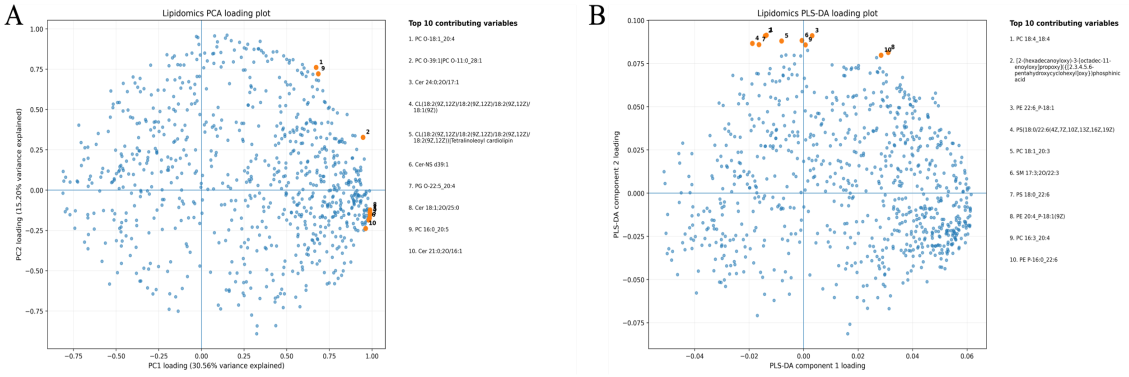

Supplementary Figure S3. Loading plots of the lipidomic multivariate analyses.

(A) PCA loading plot showing the lipid variables contributing to the separation among AA, Chengkou, and Langshan chickens. (B) PLS-DA loading plot showing the lipid variables contributing to discrimination among the three breeds. The numbered points indicate the top 10 contributing variables.
