# Supplementary material for: Comparative Analysis of Volatile Compounds, Amino Acids, Fatty Acids, and Lipidomic Profiles in Thigh Muscles of Commercial Arbor Acres (AA) Broilers and Indigenous Chengkou and Langshan Chickens
Source: Foods. 2026 Aug 31;15(17):3098. doi: 10.3390/foods15173098 (PMC13565686; doi:10.3390/foods15173098)
Supplement: Supplementary file 1 [file foods-15-03098-s001.zip › Supplementary Figure S4.pdf]

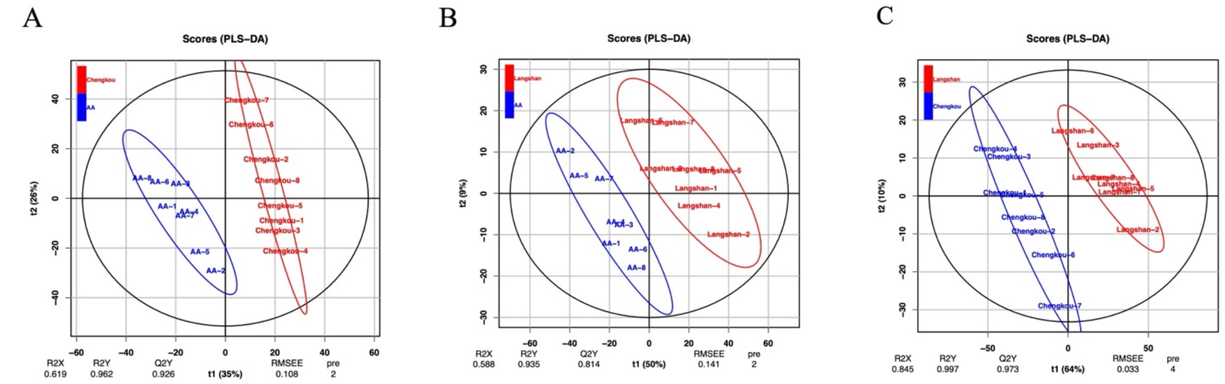

Figure S4. Permutation test validation of PLS-DA models based on lipidomic profiles.

(A) AA broilers versus Chengkou chickens; (B) AA broilers versus Langshan chickens;(C) Chengkou chickens versus Langshan chickens. A total of 200 permutations were performed to evaluate model robustness and potential overfitting.
