# Supplementary material for: Comparative Analysis of Volatile Compounds, Amino Acids, Fatty Acids, and Lipidomic Profiles in Thigh Muscles of Commercial Arbor Acres (AA) Broilers and Indigenous Chengkou and Langshan Chickens
Source: Foods. 2026 Aug 31;15(17):3098. doi: 10.3390/foods15173098 (PMC13565686; doi:10.3390/foods15173098)
Supplement: Supplementary file 1 [file foods-15-03098-s001.zip › Supplementary Table S1.pdf]

Table S1. Feed Formulation and Nutritional Levels of Experimental Chickens

Ingredients/Nutritional Indicators	Langshan and Chengkou Mountainous Chickens		Arbor Acres (AA) Broilers	
	0~3 weeks of age (516)	4~Market (526)	0~3 weeks of age (516H)	4~Market (526H)
Corn (g/kg)	510	540	470	430
Soybean Meal (g/kg)	320	240	350	310
Flour (g/kg)	100	100	100	100
DDGS (g/kg)	—	30	—	30
Glutamate Residue (g/kg)	10	10	—	10
Oil (g/kg)	10	30	20	70
Premix (g/kg)	50	50	50	50
Total (g/kg)	1000	1000	1000	1000
Metabolizable Energy (kcal/kg)	2850	3100	2950	3200
Crude Protein (%)	21	19	21.0-24.0	19.0-22.0
Crude Fiber (%)	⩽6.5	⩽6.5	⩽6.5	⩽6.5
Crude Ash (%)	⩽10.2	⩽10.5	⩽10.2	⩽10.5
Calcium (%)	0.5-1.5	0.5-1.56	0.5-1.56	0.5-1.56
Total Phosphorus (%)	⩾0.30	⩾0.30	⩾0.30	⩾0.30
Sodium Chloride (%)	0.2-0.9	0.2-0.9	0.2-0.9	0.2-0.9
Lysine (%)	⩾1.1	⩾0.9	⩾0.95	⩾0.9
Moisture (%)	⩽13.5	⩽13.5	⩽13.5	⩽13.5
