# Supplementary material for: Comparative Analysis of Volatile Compounds, Amino Acids, Fatty Acids, and Lipidomic Profiles in Thigh Muscles of Commercial Arbor Acres (AA) Broilers and Indigenous Chengkou and Langshan Chickens
Source: Foods. 2026 Aug 31;15(17):3098. doi: 10.3390/foods15173098 (PMC13565686; doi:10.3390/foods15173098)
Supplement: Supplementary file 1 [file foods-15-03098-s001.zip › Supplementary Table S2.pdf]

Table S2. Full list of KEGG pathway enrichment results for differential lipids in chicken thigh muscle.

Metabolic pathway	Total lipids in pathway	Expected hits	Differential lipids (hits)	Raw P value	-log10 (*P* value)	Holm-adjusted *P* value	FDR (Benjamini–Hochberg)	Pathway impact score
Glycerophospholipid metabolism	36	0.55608	12	6.0395E-15	14.219	4.8316E-13	4.8316E-13	0.46667
Sphingolipid metabolism	32	0.49429	4	0.0011697	2.9319	0.092409	0.042217	0.31332
Glycerolipid metabolism	16	0.24715	3	0.0015832	2.8005	0.12349	0.042217	0.02648
Linoleic acid metabolism	4	0.061786	1	0.060429	1.2188	1	1	0
Glycosylphosphatidylinositol (GPI)-anchor biosynthesis	31	0.47884	2	0.080944	1.0918	1	1	0.03672
Arachidonic acid metabolism	41	0.63331	2	0.13017	0.88548	1	1	0.02219
alpha-Linolenic acid metabolism	13	0.20081	1	0.18388	0.73545	1	1	0
Ether lipid metabolism	17	0.26259	1	0.23363	0.63148	1	1	0.19188
Inositol phosphate metabolism	30	0.4634	1	0.37603	0.42477	1	1	0.07332
Steroid biosynthesis	41	0.63331	1	0.47641	0.32202	1	1	0
Primary bile acid biosynthesis	46	0.71054	1	0.51675	0.28672	1	1	0.03799
Steroid hormone biosynthesis	74	1.143	1	0.69315	0.15917	1	1	0
