# Supplementary material for: Comparative Analysis of Volatile Compounds, Amino Acids, Fatty Acids, and Lipidomic Profiles in Thigh Muscles of Commercial Arbor Acres (AA) Broilers and Indigenous Chengkou and Langshan Chickens
Source: Foods. 2026 Aug 31;15(17):3098. doi: 10.3390/foods15173098 (PMC13565686; doi:10.3390/foods15173098)
Supplement: Supplementary file 1 [file foods-15-03098-s001.zip › Supplementary Table S3.pdf]

Table S3. Fatty acid nutritional indices of thigh muscle from different chicken breeds.

Nutritional index	AA chicken	Chengkou chicken	Langshan chicken
PUFA/SFA	0.862	1.079	0.831
n-6/*n*-3 PUFA ratio[1]	14.77	13.970	23.95
Index of atherogenicity (IA)	0.340	0.322	0.343
Index of thrombogenicity (IT)	0.905	0.827	1.004
Hypocholesterolemic/hypercholesterolemic ratio (HH)	2.569	2.841	2.580
Health-promoting index (HPI)	2.939	3.104	2.919

Note: Nutritional indices were calculated using the breed-group mean fatty acid contents reported in Table 2 (based on n = 6 biological replicates per breed). C12:0, C14:0, and C22:5n3 (DPA) were absent from the quantified fatty acid dataset and therefore contributed zero to the calculations. [1]C20:2 (eicosadienoic acid) was classified as an *n*-6 polyunsaturated fatty acid (C20:2n6) and included in the calculation of Σ*n*-6 PUFA for the *n*-6/*n*-3 ratio and IT index. IA and IT were calculated according to Ulbricht and Southgate (1991), HH according to Santos-Silva et al. (2002), and HPI according to Chen et al. (2004).

PUFA/SFA = ΣPUFA / ΣSFA;
n-6/*n*-3 = Σ*n*-6 PUFA / Σ*n*-3 PUFA;
IA = [C12:0 + (4 × C14:0) + C16:0] / ΣUFA;
IT = (C14:0 + C16:0 + C18:0) / [(0.5 × ΣMUFA) + (0.5 × Σ*n*-6 PUFA) + (3 × Σ*n*-3 PUFA) + (Σ*n*-3 PUFA / Σ*n*-6 PUFA)];
HH = (C18:1n9c + C18:2n6c + C18:3n3 + C20:4n6 + C20:5n3 + C22:6n3) / (C14:0 + C16:0);
HPI = ΣUFA / [C12:0 + (4 × C14:0) + C16:0].
